# Supplementary material for: Morphology and calcification characterization in patients undergoing TAVI: A 3D statistical shape modelling study
Source: PLOS Digit Health. 2025 Jul 21;4(7):e0000564. doi: 10.1371/journal.pdig.0000564 (PMC12279150; doi:10.1371/journal.pdig.0000564)
Supplement: S1 Fig — Variables are divided between binary and continuous variables. The relation between two variables was assessed by respectively: a Pearson’s chi-squared test for two binary variables, a t-test for one binary and one continuous variables, and a Pearson’s correlation test for two continuous variables. (PDF) [file pdig.0000564.s001.pdf]

Supporting figure S1_Fig to the article *Morphology and calcification characterization in patients undergoing TAVI: A 3D Statistical Shape Modelling Study* by Sivera et al. 2025.

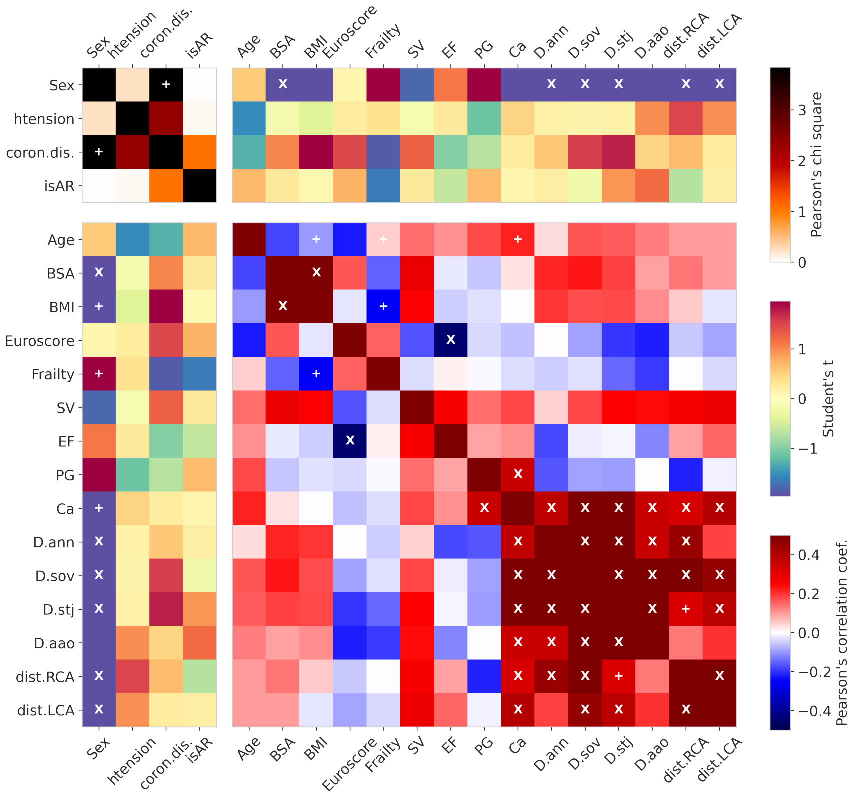

Figure 1. Correlation matrix of the pairwise analysis of the clinical data. Variables are divided between binary and continuous variables. The relation between two variables was assessed by respectively: a Pearson's chi-squared test for two binary variables, a t-test for one binary and one continuous variables, and a Pearson's correlation test for two continuous variables. White x indicates a significant result for Bonferoni's correction ($p < 0.05/(N(N-1)/2) \approx 2.9 \cdot 10^{-4}$), + indicates a significant result for a false discovery rate (FDR) of 1% ($p < 0.01$).
