# Supplementary figures and images for: Morphology and calcification characterization in patients undergoing TAVI: A 3D statistical shape modelling study
Source: PLOS Digit Health. 2025 Jul 21;4(7):e0000564. doi: 10.1371/journal.pdig.0000564 (PMC12279150; doi:10.1371/journal.pdig.0000564)

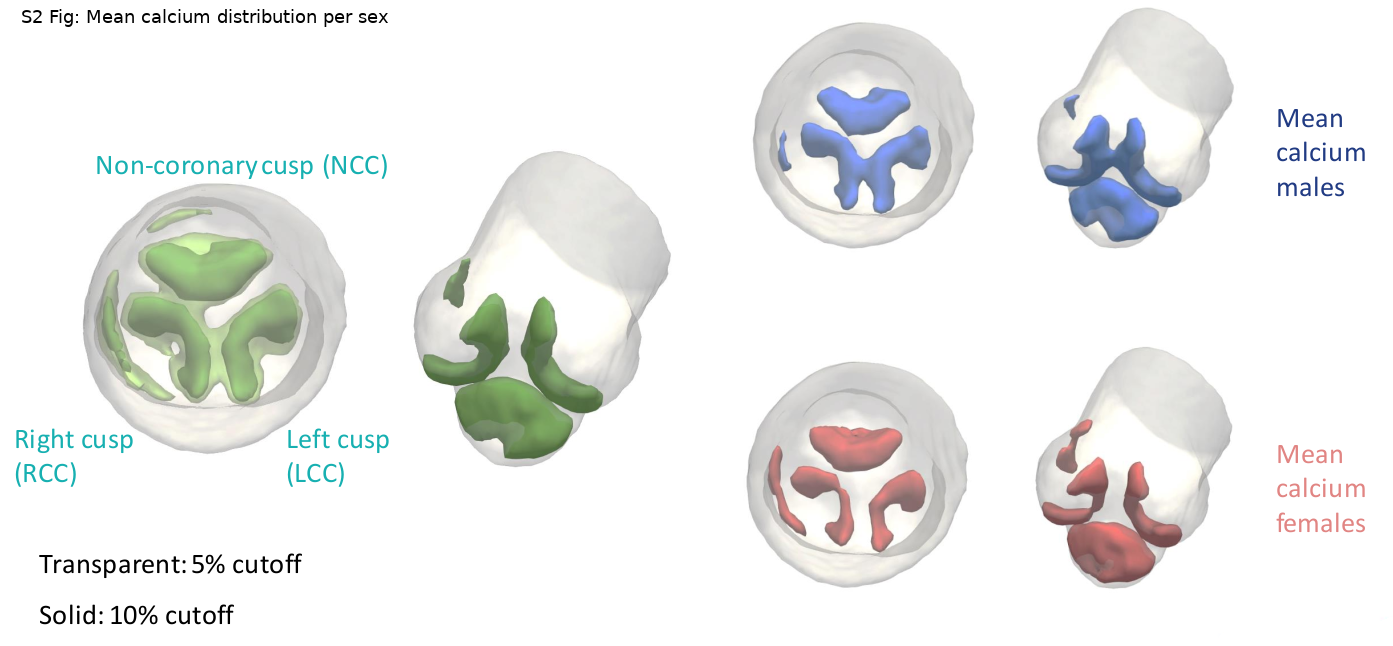

Supplement: S2 Fig — 3D visualization of the average calcium aggregates for the whole population and both genders separately. (PNG) [file pdig.0000564.s002.png]

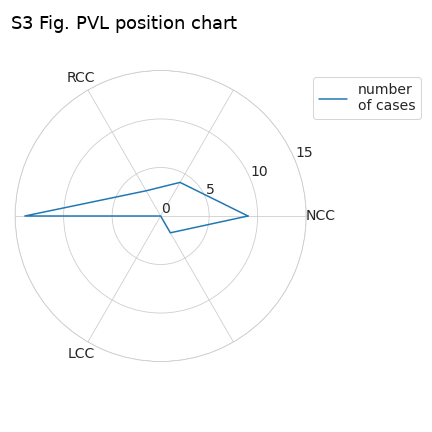

Supplement: S3 Fig — Visualization of the paravalvular leakage position for the 26 cases where it has been reported in our population. (PNG) [file pdig.0000564.s003.png]

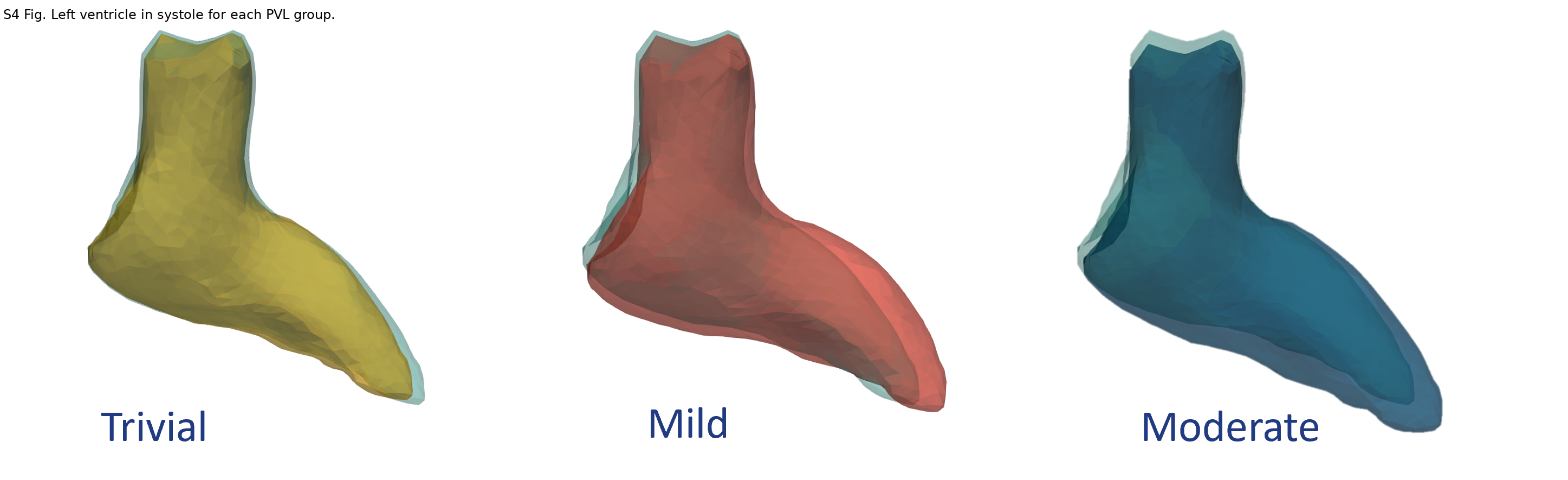

Supplement: S4 Fig — (PNG) [file pdig.0000564.s004.png]
