# Supplementary material for: Morphology and calcification characterization in patients undergoing TAVI: A 3D statistical shape modelling study
Source: PLOS Digit Health. 2025 Jul 21;4(7):e0000564. doi: 10.1371/journal.pdig.0000564 (PMC12279150; doi:10.1371/journal.pdig.0000564)
Supplement: S1 Appendix — Probabilistic modelling of the local calcium distribution and comparison to empirical results. (PDF) [file pdig.0000564.s005.pdf]

Supporting information S1 to the article *Morphology and calcification characterization in patients undergoing TAVI: A 3D Statistical Shape Modelling Study* by Sivera et al. 2025.

Calcification density Bayesian modelling

The statistical analysis of the calcium density maps relies on a probabilistic modelling of the density at each voxel. We do not aim for an exact modelling but an approximate description allows for a better comparison of different subjects, at different locations, with highly variable density patterns. The original calcium segmentations are, primarily, binary. However, realignment, smoothing, and re-slicing lead to continuous measurements of local calcium density. The calcium intensity on the 2D cross-sectional slice is modelled using a the product of Bernoulli distribution and of a gamma distribution to take into account the accumulation of calcium at variable rates at multiple positions. For every pixel j

$$x_j \sim B(p_j) \cdot \Gamma(k_j, \theta_j)$$

We focus here on the modelling of single pixels and, consequently, the pixel indexing (j) is ignored in the following. We present here results for the valve slice. Pixels with very low density (null or below 0.023 (arbitrarily chosen such that the sum over the population is lower than 3)) are ignored, resulting in 670 included pixels. The samples (i.e. the subjects) are indexed by $i \in [1, n]$. We note $\overline{y}$ the empirical average of a variable y. The maximum of likelihood estimators of the parameters p, k, θ can be approximated by:

$$\hat{p} = \text{card}\{i, x_i == 0\}/n$$
$$\hat{\theta} = \overline{x \log(x)} - \overline{x}\overline{\log(x)}$$
$$\hat{k} = \overline{x}/\hat{\theta}$$

Figure **??**a shows 6 examples corresponding to various average density. We see on the first row the maximum log-likelihood curve for varying θ. The optimized $\hat{\theta}$ is represented by the vertical dashed line. We can see that small variation of θ, in particular towards higher values, has a minimal impact of the maximum likelihood. The second row shows the fitter probability density function and the histogram of observed values. It illustrates the difficulty to fit the distribution at each point and the importance of the threshold used to numerically define 0 and > 0 to account for the high frequency of no-calcification. The distribution of the estimated parameters is shown in Figure **??**b. We observe that k and p are correlated. It reflects the fact that regions that are rarely calcified, are also not highly calcified in patients in which they are affected. Regarding θ, this result highlights the higher variability of the estimator in low calcification area (low k and high p). This variability can be both associated to the underlying calcification pattern and to the limit of the estimator. Finally Figure **??**c reports the global Bayesian information criteria (BIC) difference between the models with fixed θ, and the base model where θ is optimized at each pixel. The BIC is defined by:

$$\text{BIC} = k \log(n) - 2 \log(\hat{L})$$

where $\hat{L}$ is the maximized value of the likelihood, $n = n_{\text{pixels}} \times n_{\text{subjects}}$ the number of data points, and k the number of parameters. These three results argue for a choice fixed value of θ to limit the freedom of the model, to regularized the comparison between several subgroups. A value of $\theta = 2.2$ has been chosen, near the BIC optimum. A safely larger value is expected to lead to more stable results.

This analysis does not fully answer the problem of calcium modelling. In particular, the most important question, the spatial patterns, is not considered here. We however argue that this modelling allows for the reasonable comparison of multiple 3D calcification patterns between several groups at a voxel level.

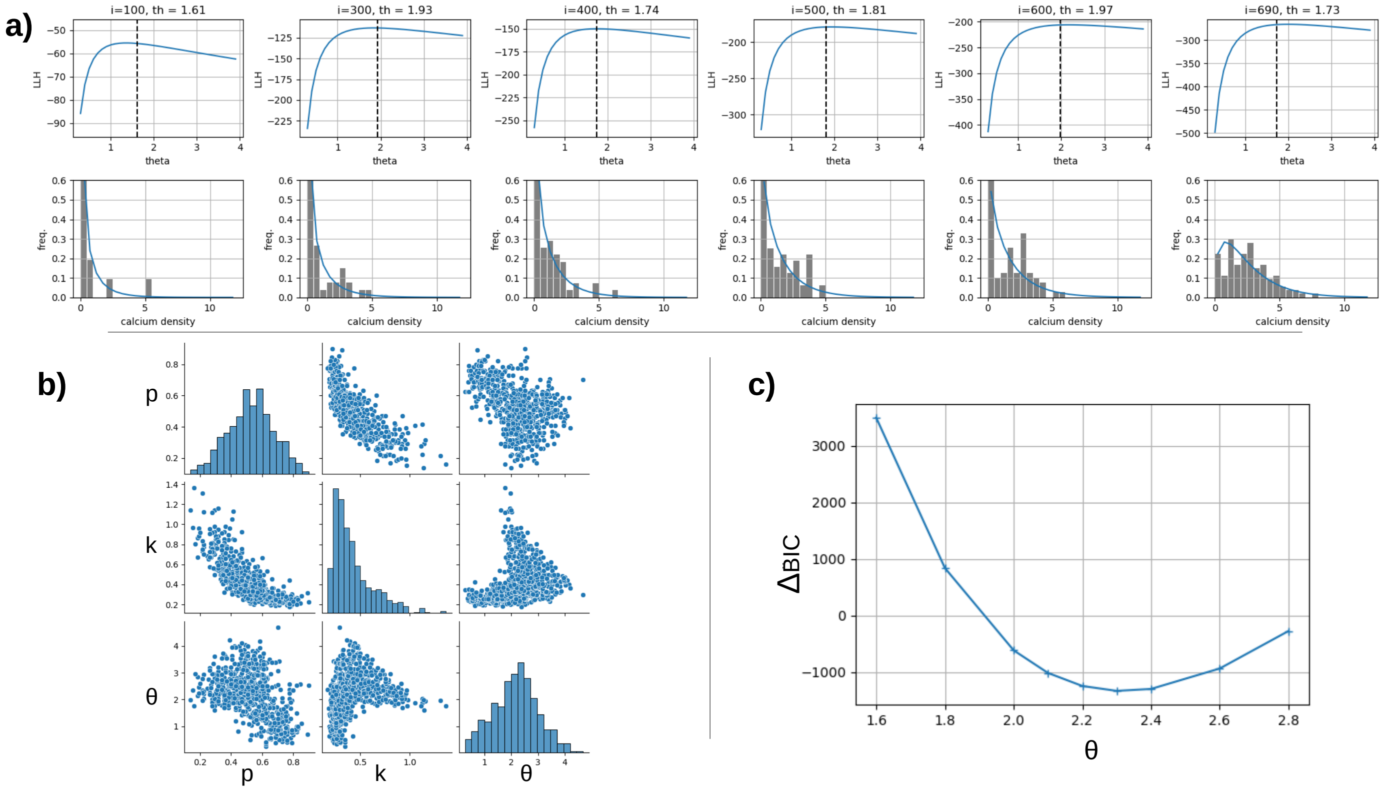

Figure 1. Optimization of calcium density probabilistic parameters. a) LLH curves (top) and fitted distribution (bottom row) for 6 pixels with increasing average density from left to right. b) Fitted parameters for the 670 pixels: histograms and paired distributions. c) BIC difference between models with a fixed value of θ and the model where θ is optimized at each voxel, lower BIC indicates a better balance between model fit and number of parameters.
