# Supplementary material for: Morphology and calcification characterization in patients undergoing TAVI: A 3D statistical shape modelling study
Source: PLOS Digit Health. 2025 Jul 21;4(7):e0000564. doi: 10.1371/journal.pdig.0000564 (PMC12279150; doi:10.1371/journal.pdig.0000564)
Supplement: Attachment — Submitted filename: PDIG-D-24-00265_reviewer_JULIANBORGES_FINAL.pdf [file pdig.0000564.s006.pdf]

PDIG-D-24-00265

Enriching the characterization of patients undergoing TAVI: what can the aortic morphology and calcification tell us?

Research Article

Authors: Raphael Sivera; Ebba Montgomery-Liljeroth; Yaxi Chen; Silvia Schievano; Jan Brüning; Wouter Huberts; Anthony Mathur; Andrew Cook; Kush Patel; Claudio Capelli

Reviewer: Julian Borges

Overview

This study presents a novel approach to characterizing patients undergoing Transcatheter Aortic Valve Implantation (TAVI) using advanced statistical shape modeling (SSM) and 3D imaging analysis. It identifies associations between aortic morphology, calcification patterns, and procedural outcomes, notably paravalvular leakage (PVL). These findings have potential implications for clinical practice, device design, and pre-operative planning in cardiovascular care.

Major Issues

1. **Methodological Clarity in Calcification Measurement**
   - **Issue**: The study relies on manual segmentation and thresholding for calcification measurement, which can introduce subjectivity and variability.
   - **Recommendation**: Clarify the segmentation process and threshold choice in the Methods section. If possible, provide details on inter-operator reliability or consistency checks. Consider discussing alternative semi-automated or AI-based approaches that could enhance reproducibility.
2. **Control for Confounding Variables**
   - **Issue**: Limited adjustments are made for confounding factors such as comorbidities, medications, and demographic variations (e.g., age and sex) that could impact outcomes.
   - **Recommendation**: Strengthen the analysis by incorporating adjustments for these factors, either through multivariable regression models or by providing stratified analysis (e.g., by sex or device type). This will better isolate the impact of morphology and calcification on PVL outcomes.
3. **Statistical Rigor and Reporting**
   - **Issue**: While the study identifies associations, it lacks detailed reporting on effect sizes, confidence intervals, and the statistical power of analyses.

- **Recommendation**: Report effect sizes alongside p-values to provide context on the practical significance of findings. Additionally, if the study was underpowered for certain analyses, this should be acknowledged in the limitations.

4. **Utility and Practical Implications**
   - **Issue**: The study provides limited actionable insights for clinicians or device manufacturers regarding how findings might influence TAVI practice or device selection.
   - **Recommendation**: Expand on how specific morphological or calcification traits could guide device choice or procedural planning. Consider adding a brief discussion on future research directions, such as the development of sex-specific devices.
5. **Data Accessibility and Open Science Standards**
   - **Issue**: The study mentions data availability but does not provide direct access to anonymized imaging data or segmentation files, which could hinder reproducibility.
   - **Recommendation**: If possible, make de-identified imaging data or shape models accessible to support open science practices, or include a more detailed data availability statement.

Minor Issues

1. **Title Improvement**
   - **Recommendation**: Consider a more descriptive title, such as: *"Morphological and Calcification Predictors of Outcomes in TAVI Patients: A 3D Statistical Shape Modeling Study."* This better conveys the focus on outcomes and the modeling approach.
2. **Detailed Methods for Statistical Shape Modeling (SSM)**
   - **Issue**: The process for selecting control points and kernel parameters in SSM is not fully explained, potentially impacting reproducibility.
   - **Recommendation**: Include additional detail on parameter choices and optimization steps within the Methods section or in supplementary materials.
3. **Expand on Sexual Dimorphism**
   - **Recommendation**: Sexual dimorphism is discussed, but the underlying mechanisms remain speculative. If possible, explore correlations with specific biological factors (e.g., hormone levels or tissue properties) or acknowledge this as an area for further research.
4. **Clarify Use of Open Science Tools**
   - **Issue**: Open science practices are briefly mentioned but could be expanded.
   - **Recommendation**: List specific repositories or platforms where data could be hosted (e.g., Dryad, OSF) and provide a rationale for data sharing practices.

Other Comments

- **Figures and Tables**: Figures are clear, well-labeled, and informative. They effectively illustrate key morphological features and calcification patterns, though improved annotations or cross-sectional views of calcification in high-risk areas (e.g., coronary cusps) might further enhance clarity.
- **Methods**: While generally well-detailed, certain sections (e.g., SSM parameterization, calcification segmentation) could benefit from additional specificity to improve reproducibility and transparency.
- **Study Design**: The design is appropriate for the research question, given its retrospective nature, but future prospective or multi-center validation would strengthen the findings and broaden applicability.
- **Experiments**: All relevant experiments for a retrospective study appear to be included. However, if data on additional anatomical features, such as fibrotic tissues or coronary anatomy, are available, these could be considered in future analyses.
- **Statistics**: The statistical analyses are generally sound but would benefit from the inclusion of effect sizes and confidence intervals for associations. Additionally, detailing statistical power for key analyses would provide insight into the robustness of findings.
- **Results and Data**: The results support the conclusions, but they would be strengthened by further adjustments for confounding variables and reporting of effect sizes. Accessibility to primary data (e.g., anonymized imaging) would align with open science practices.
- **References**: Comprehensive and relevant references are included. No major omissions identified.

This study offers novel insights into the TAVI procedure, linking patient-specific anatomy with procedural outcomes. Highlighting it on the *PLOS Digital Health* website could raise awareness within the digital health and cardiovascular communities, facilitating future work that builds on these findings.
