# Supplementary material for: Morphology and calcification characterization in patients undergoing TAVI: A 3D statistical shape modelling study
Source: PLOS Digit Health. 2025 Jul 21;4(7):e0000564. doi: 10.1371/journal.pdig.0000564 (PMC12279150; doi:10.1371/journal.pdig.0000564)
Supplement: Attachment — Submitted filename: reviews_response_TAVI.pdf [file pdig.0000564.s007.pdf]

We thank both Reviewer 1 and Reviewer 2 for their extensive and positive feedback on our work. We believed that their remarks improved the clarity and reproducibility of our study, but also its potential impact. We detail below our response to their comments and suggestions, point by point.

Reviewer #1:

Major Issues

I.1. Methodological Clarity in Calcification Measurement
Issue: The study relies on manual segmentation and thresholding for calcification measurement, which can introduce subjectivity and variability.
Recommendation: Clarify the segmentation process and threshold choice in the Methods section. If possible, provide details on inter-operator reliability or consistency checks. Consider discussing alternative semi-automated or AI-based approaches that could enhance reproducibility.

We acknowledge the recommendation of Reviewer 1 and we here provide more information about the segmentation process for identifying and measuring the volumes of calcification from all the patients' images. The Reviewer is indeed correct that the process of segmentation was mainly based on a semi-automatic process which started from the thresholding operation and continued with manual corrections performed on each slide of the datasets where calcium was identified. The thresholding values for the population varied between 300 and 1000 Hounsfield Unit. For consistency, the segmentations were performed by a single operator (YC), following appropriate training, and reviewed by CC with more than 15 years of experience in segmentation. We also agree with the Reviewer that this process will benefit more automatic, AI-based approaches and we added such remark in the Discussion Section.

ORIGINAL – 5.3 Methods -- Image processing

Calcific tissues were segmented by manually thresholding the image and delineating the calcified area. A base threshold of 300 Hounsfield unit was adapted to each image based on the operator assessment. We stress the fact that the focus of the study was on the spatial description of the calcification pattern and that better segmentations were favoured over consistent estimation of calcification scores.

AMENDED – 5.3 Methods -- Image processing

Calcifications at the implantation site were segmented by a semi-automatic process consisting of initial image thresholding to identify the calcified area followed by manual refinements performed on a slice-by-slice basis. Thresholding was adjusted between 300 and 1000 Hounsfield unit, depending on the characteristics of each dataset. To maintain consistency across segmentations, these were performed by a single, trained operator and subsequently reviewed by an experienced member of the research team. The

resulting segmentations provided a detailed spatial representation of the calcification distribution and enabled quantitative assessment of calcification volumes.

ORIGINAL – 3 Discussion – Study limitations

(...) The segmentation of the calcium was challenging because of image artefacts near very bright aggregates.

AMENDED – 3 Discussion – Study limitations

The segmentation of the calcium was challenging because of image artefacts near very bright aggregates necessitating multiple manual inspections and corrections by at least two independent operators. This process was time consuming. In future studies, these limitations are expected to be mitigated through the development and application of more automated approaches, including deeplearning-based methods specifically trained on this cohort.

I.2. Control for Confounding Variables
Issue: Limited adjustments are made for confounding factors such as comorbidities, medications, and demographic variations (e.g., age and sex) that could impact outcomes.
Recommendation: Strengthen the analysis by incorporating adjustments for these factors, either through multivariable regression models or by providing stratified analysis (e.g., by sex or device type). This will better isolate the impact of morphology and calcification on PVL outcomes.

We agree with Reviewer 1 that the relation between the morphology, the calcification, and the assessed outcomes are intertwined and affected by multiple confounding factors. In particular, the interactions between size, shape, calcification and sex illustrated the difficulty to isolate specific effects (see Sections 2.4.3 and 2.4.4 and Figures 6 and 7). This is especially true for sex as it intersects with many factors (e.g. shape, effect of hormones on the vasculature, other comorbidities and lifestyle) that have the potential to affect the aorta, the atherosclerosis, and the TAVI outcome. A more complete description of the TAVI population can provide additional information to such challenge, enriching the existing knowledge with shape effect on calcification or link between shape features, device implantation and PVL for example, but the direct prediction of TAVI outcome is beyond the scope of this work.

However, we agree that the results regarding the shape correlation with PVL presented in section 2.4.5 could also benefit from a more stratified analysis. The type of device is definitely an essential factor and to this end we included results of the analysis done on the most common device. Several multilinear analyses are proposed that includes sex, annulus diameter, SoV diameter, and device size as regressors to assess the remaining relation between sex and shape, and PVL and shape. Including additional co-factors may hide or introduce new effects due to their inter-dependence.

The main findings of the additional analysis showed that:

- *including one diameter sexual dimorphism is still visible, but including both the annulus and SoV diameters, sexual dimorphism is highly reduced. The female average aorta is still visibly straighter but no significant difference is measured. The increase in r2 of 1.6% (from 0.121 to 0.137) is to be compared to the original 3.3%.*
- *including both diameters and the device size, the positive PVL average has still a longer LCC sinus, but the difference is very small (around 1mm difference) and non-significant. Including sex as a co-regressor does not affect this result.*

We do not believe it affects the study's conclusions, in particular the effect of diameters on the outcome and their association with sex dimorphism remains relevant.

As stated, we presented an exploratory analysis to identify features of the population. However, it has its limitations when it includes so many different comparisons, and calls for future additional external validation.

The section 2.4.5 was also renamed to more accurately communicate our analysis. We are indeed assessing the relation between outcomes and shape features (treating shape as the response variable) and not aiming at establishing a causal relation from shape to outcome.

ORIGINAL – 2.2.1 Results -- Shape correlations with clinical parameters -- Aortic root shape

The aortic shape also correlated with sex, pressure gradient (PG) and ejection fraction (EF), and the reported calcification volume. Weaker (non-significant) associations were also visible with age, BSA and BMI.

The relation with sex was mainly caused by size difference but dimorphism was also observed when correcting for diameter. Similarly, as seen previously, higher calcification volume was directly correlated to aorta sizes but the correlation between shape and total calcification was still visible, and significant (p=0.01), when corrected for diameter differences: bulkier, straighter aortas with longer and lower sinuses being associated with more calcification.

Finally, wider and straighter aortas were associated with higher EF, while more compact aortic root with smaller cusps and narrower sino-tubular junction were positively associated with higher PG.

AMMENDED – 2.2.1 Results -- Shape correlations with clinical parameters -- Aortic root shape

The aortic shape also correlated with sex, pressure gradient (PG) and ejection fraction (EF), and the reported calcification volume, the shape statistical effects are visualized in Figure 3. Wider and straighter aortas were associated with higher EF, while more compact aortic root with smaller cusps and narrower sino-tubular junction were positively associated with higher PG. Weaker (non-significant) associations were also visible with age, BSA and BMI but are not represented.

The relation with sex was mainly caused by size difference but dimorphism was also observed when correcting for (annulus) diameter ($p<0.01$, r2=1.9%). More precisely, female average phenotype appeared to be more cylindric while the male average has comparatively larger sinuses and is more curved. We note that when both the annulus and the SoV diameters are included as co-regressor in a multivariate regression the female average is still straighter but the overall effect of sex is reduced and not significant anymore (p=0.4, r2=1.6%).

Finally, higher calcification volume is, as seen previously, directly correlated to aortic sizes, but the correlation between shape and total calcification was still visible, and significant (p=0.01, r2=2.7%), when corrected for diameter differences: bulkier, straighter aortas with longer and lower sinuses being associated with more calcification.

ORIGINAL – 2.4.5 Results – Prediction of interventional outcomes -- Shape outcome predictors

AMMENDED – 2.4.5 Results – Prediction of interventional outcomes -- Shape association with outcomes

ADDED 2.4.5 Results – Prediction of interventional outcomes -- Shape association with outcomes

To further study the interest of the shape features, we restricted the analysis to the most common device type (SAPIEN model) and included both diameters and the device size as co-regressors. The positive (mild or plus) PVL average still had a longer LCC sinus than the control group, but the difference was small (around 1mm difference) and non-significant (p=0.5, r2=0.9%). Including sex as a co-regressor does not affect this result.

I.3. Statistical Rigor and Reporting
Issue: While the study identifies associations, it lacks detailed reporting on effect sizes, confidence intervals, and the statistical power of analyses.
Recommendation: Report effect sizes alongside p-values to provide context on the practical significance of findings. Additionally, if the study was underpowered for certain analyses, this should be acknowledged in the limitations.

Assessment of the effect size is indeed crucial to understand the relevance of observed associations. In this work, we measured the association between individual scalar variables and the modelled shapes using the linear least square r2. We also visualized resulting difference in average shape and calcification between sub-groups. Additional assessments of the effect size such as average surface distance etc. would be possible but we believe that these quantitative measurements would potentially be misleading without bringing any additional clinically relevant information compared to the 3D visualizations.

We also considered that it was beyond the scope of this work to assess directly the effect of specific (shape or calcification) features on the intervention outcome, for two main reasons. First, the choice of the features would be affected by the current exploratory analysis: an independent population would be preferable. Second, shape and calcifications regularly impact the procedural decisions, in a way that is very complex to account in a retrospective study. We agree that a future study would be needed to investigate the relevance for the clinical practice.

We took in consideration this remark in all of our amendments, but in particular we edited Section 2.2.1 as follow.

ORIGINAL – 2.2.1 Results -- Shape correlations with clinical parameters -- Aortic root shape

Potential association between selected clinical variables and the aortic shape model parameters (momenta) were independently tested. Results are presented in Table 2. The strong relationship with aortic diameters is a validation of the methodology but also give a quantitative reference to analyse further results. The aortic shape also correlated with sex, pressure gradient (PG) and ejection fraction (EF), and the reported calcification volume.

AMMENDED – 2.2.1 Results -- Shape correlations with clinical parameters -- Aortic root shape

Potential association between selected clinical variables and the aortic shape model parameters (momenta) were independently tested. Results are presented in Table 2. First, the strong relationship with aortic diameters is a validation of the methodology but also give a quantitative reference to analyse further results. The effect size is here primarily quantified by the linear regression r2 and, in the case of the individual diameters the value range from 0.07 for the annulus to 0.13 for the StJ; meaning that these single explanatory variables were able to account, respectively, for 7 or 13% of the total variance in the shape model. The aortic shape also correlated with sex, pressure gradient (PG) and ejection fraction (EF), and the reported calcification volume, the shape statistical effects are visualized in Figure 3. Wider and straighter aortas were associated with higher EF, while more compact aortic root with smaller cusps and narrower sino-tubular junction were positively associated with higher PG.

I.4. Utility and Practical Implications
Issue: The study provides limited actionable insights for clinicians or device manufacturers regarding how findings might influence TAVI practice or device selection.
Recommendation: Expand on how specific morphological or calcification traits could guide device choice or procedural planning. Consider adding a brief discussion on future research directions, such as the development of sex-specific devices.

We acknowledge the Reviewer's comment that the insights for clinicians or device manufacturers might not appear too direct and rather focused on the morphological description of patients undergoing TAVI. However, the enriched morphological analysis can speak to clinicians (to predict outcomes), device manufacturers (to consider specific features of the implantation site) or both to conduct stratified statistical analysis including the so-called in silico trials. This can be particularly relevant if the morphological characterization takes into account a wider range of factors – such as, for example. the complexity of inter-individual variability and the presence of relevant comorbidities.

We agree with the Reviewer that this is mainly a future direction and, therefore, a specific point of discussion has been added to the Discussion section.

ORIGINAL – Abstract

In conclusion, the presented morphological characterization of patients with AS could contribute to understand TAVI outcomes and plan future improvement of TAVI device design.

AMENDED – Abstract

In the future, the presented morphological characterization of patients with AS could contribute to predict post-TAVI PVL and design and test improved TAVI devices.

ORIGINAL – Discussion -- Morphological description of AS patients

Such characterization might enrich the generation of synthetic AS geometries as the ones recently proposed by Verstraeten et al. (2024) for in-silico clinical trials.

AMENDED -- Discussion -- Morphological description of AS patients

Such enriched characterization might contribute to a more informed generation of detailed models of AS geometries for in-silico clinical trials - such as the one recently proposed by Verstraeten et al. (2024). Indeed, the SSM of the aorta and LVOT, the associated calcifications, and their relations to demographic and clinical variables, can be used to generate synthetic anatomies that reflect the morphological variability observed in a real patient population, helping both the design and testing of TAVI devices. The identified morphological traits (size, diameters, sinus and LVOT shape, calcium distribution) can also suggest features of importance for procedural planning, stressing common patterns of calcification that could impede proper valve expansion and sealing and would require specific care. Future studies will focus on quantifying the presented relationships and incorporating them into clinical decision-making processes and computational testing.

I.5. Data Accessibility and Open Science Standards
Issue: The study mentions data availability but does not provide direct access to anonymized imaging data or segmentation files, which could hinder reproducibility.

Recommendation: If possible, make de-identified imaging data or shape models accessible to support open science practices, or include a more detailed data availability statement.

We acknowledge the Reviewer's comment. Access to de-indentified data is available through the SIMCor Virtual Research Environment (VRE) - https://simcor.unitbv.ro/vre/. The platform can be accessed by interested users who can register for R&D purpose only. This information has been added to the manuscript.

ADDED – Data availability

Access to de-indentified data is available through the SIMCor Virtual Research Environment (VRE) - https://simcor.unitbv.ro/vre/. The platform can be accessed by interested users who can register for R&D purpose only.

Minor issues

I.6. Title Improvement: Consider a more descriptive title, such as: "Morphological and Calcification Predictors of Outcomes in TAVI Patients: A 3D Statistical Shape Modelling Study." This better conveys the focus on outcomes and the modeling approach.

We agree that the title could better conveys our objectives and approach, we however do not think it should focus on outcome predictions, we opted for: Morphology and calcification characterization of patients undergoing TAVI: A 3D Statistical Shape Modelling Study

---

I.7. Detailed Methods for Statistical Shape Modeling (SSM)
Issue: The process for selecting control points and kernel parameters in SSM is not fully explained, potentially impacting reproducibility.
Recommendation: Include additional detail on parameter choices and optimization steps within the Methods section or in supplementary materials.

We agree with the Reviewer that more details were needed in the method description to improve the reproducibility of the shape model estimation. Thus, we included an additional paragraph describing the process of selecting the model parameters, and the selected values. In addition to publishing the model on the online repository, we believe this will make the analysis more reproducible.

ADDED – 5.4 Methods – SSM framework:

The choice of the kernel widths, and of a good initialization, can be challenging. Heuristics have been published to guide this process (Bruse2016) but the decisions often remain subjective. To circumvent this issue, we rely on the assessment of the

reconstruction accuracy to guarantee that the model was able to capture the morphological variability in the population and that the optimization was successful.

In this work, experiments were first conducted using different kernel sizes (from 5mm to 23mm) on a random subsample of the population and a fixed number of iterations. The deformation kernel width was selected based on the maximization of the model's likelihood (after visual inspection of the result to check that no subject was showing aberrant results). The shape kernel width was selected to minimize the surface reconstruction error.

The selected parameters were λW = 7.0mm, λV = 11.0mm, and λN = 2.0 for the aorta, and λW = 5.0mm, λV = 11.0mm, and λN = 2.0 for the left ventricle. Control points were distributed in a regular grid over the joint bounding box that include every geometry, with 1mm margin.

I.8. Expand on Sexual Dimorphism
Recommendation: Sexual dimorphism is discussed, but the underlying mechanisms remain speculative. If possible, explore correlations with specific biological factors (e.g., hormone levels or tissue properties) or acknowledge this as an area for further research.

The topic of physical and physiological mechanisms underlying the observed sexual dimorphism is an interesting ongoing research field. It goes beyond the scope of our work, that aims to be very descriptive, but we added some possible hypotheses, highlighting the observed higher fibrosis of female patients and the known hormonal effects on vessel properties and remodelling, and references for further discussion.

ORIGINAL Discussion -- Relationship between morphology and demographic data.

The spatial variation of calcification pattern may contribute to explain the difference in severity observed between sexes for identical valve calcification (Aggarwal et al., 2013; Patel et al., 2023) but, statistically correlated with multiple factors, assessment of sex-differences causality cannot easily be summarized.

AMMENDED Discussion -- Relationship between morphology and demographic data.

The morphological differences and the spatial variation of calcification pattern may contribute to explain the difference in severity and hemodynamic response observed between sexes for identical valve calcification, and complement other identified sex-related differences such as the level of tissue fibrosis (Aggarwal et al., 2013; Chaker2017; Patel et al., 2023). Potential explanations, including hormonal effect or difference in cohorts, of the calcification differences have been proposed (DesJardin2022) but the study of their interactions with other comorbidities, risk factors, disease presentation,

diagnosis, and treatment remains a challenging and important research area that suffer from lack of data (Norris2020; DesJardin2022; Trongtorsak2023).

I.9. Clarify Use of Open Science Tools
Issue: Open science practices are briefly mentioned but could be expanded.
Recommendation: List specific repositories or platforms where data could be hosted (e.g., Dryad, OSF) and provide a rationale for data sharing practices.

As specified in the answer to Question 5, Access to de-indentified data is available through the SIMCor Virtual Research Environment (VRE) - https://simcor.unitbv.ro/vre/. The platform can be accessed by interested users who can register for R&D purpose only. This information has been added to the manuscript.

Other Comments

I.10. Figures and Tables: Figures are clear, well-labeled, and informative. They effectively illustrate key morphological features and calcification patterns, though improved annotations or cross-sectional views of calcification in high-risk areas (e.g., coronary cusps) might further enhance clarity.

Annotations of Figure 2 (showing the average calcification pattern) have been (slightly) improved.

I.11. Methods: While generally well-detailed, certain sections (e.g., SSM parameterization, calcification segmentation) could benefit from additional specificity to improve reproducibility and transparency.

We thank the reviewers for their feedback on this topic and hope that our corrections improved our work clarity and reproducibility.

I.12. Study Design: The design is appropriate for the research question, given its retrospective nature, but future prospective or multi-center validation would strengthen the findings and broaden applicability.

We fully share this opinion, this is a limitation of the study and we are looking forward to see future work in this domain.

ORIGINAL – Discussion – study limitations

The conclusions of our study are based on a cohort of patients retrospectively recruited from a single centre which might not fully representative of the AS population undergoing TAVI. In this context, there is not a homogeneous distribution of TAVI devices commonly used to perform the intervention.

AMMENDED – Discussion – study limitations

The conclusions of our study are based on a cohort of patients retrospectively recruited from a single centre which might not fully be representative of the AS population undergoing TAVI. In particular, the focus of this study was not on comparative device performance, or studying the most recent devices improvements; and in this context, there was a heterogeneous distribution of TAVI devices commonly used to perform the intervention in particular self-inflating devices are poorly represented in our sample (only 17/110, 15.5%). A wider multi-centre study covering more recent devices would provide additional insights.

I.13. Experiments: All relevant experiments for a retrospective study appear to be included. However, if data on additional anatomical features, such as fibrotic tissues or coronary anatomy, are available, these could be considered in future analyses.

Indeed, it would be interesting to analyse the fibrotic tissue (even if it can be difficult to identify it with certainty on the CTA) and the coronary anatomy. We included a comment in the discussion regarding additional data available to complement other additions.

ADDED: Discussion -- Study limitations.

Additional information could already be collected for the Doppler images (flow pattern and vessel dynamic), the CTA images (coronary anatomy and fibrotic tissue for example), and the operative reports (valve malpositioning and other complications).

I.14. Statistics: The statistical analyses are generally sound but would benefit from the inclusion of effect sizes and confidence intervals for associations. Additionally, detailing statistical power for key analyses would provide insight into the robustness of findings.

Addressed in point I.2.

I.15. Results and Data: The results support the conclusions, but they would be strengthened by further adjustments for confounding variables and reporting of effect sizes. Accessibility to primary data (e.g., anonymized imaging) would align with open science practices.

Addressed in point I.3 and I.5.

I.16. References: Comprehensive and relevant references are included. No major omissions identified.

No change required.

Reviewer #2:

II.1. CTA imaging was acquired and processed at best-diastole. **The lack of CTA imaging at best-systole should be acknowledged as a limitation** of the study. Nonetheless, exploring the impact of dynamic aortic root variations between systole and diastole on the presented analysis could provide valuable insights. The authors are encouraged to elaborate further and discuss this aspect.

Images at best-systole were also acquired but we chose to analyse the best-diastole because the valves were more visible and easier to segment. 30 systole images were however segmented for comparison purpose. No major differences (in anatomy or segmented calcification) was observed but the analysis was not systematic, nor quantified. We agree that studying the impact of the aortic motion on TAVI but also its interactions with calcification (motion induced tissue stress, stiffening, etc.) would be interesting but we believe it is a bit beyond the scope of this work. The following paragraph is added to discussion /limitations to present this perceptive.

ADDED – Discussion – Study limitations

More generally, the dynamic aortic root variation during the cardiac cycle was not studied in this work. Exploring its impact on calcification and TAVI outcome could provide valuable insights. First, tissue stress has an impact on the calcification process (Bogdanova et al. 2022) and it can be estimated based on the deformation between the best-diastole images and the best-systole images that have also been acquired for every patient. Second, the implanted devices are known to be stiffer than the native tissue and the dynamic motion may be related to PVL or conductive impairments. It should however be highlighted that calcified aortas in aging patient are quite stiff and the motion limited.

II.2. The authors are to be commended for their transparency in reporting and summarizing the limited impact of SSM-based predictors on TAVI procedural outcomes, such as PVL and pacemaker implantation. **From a clinical perspective, the authors are encouraged to elaborate on whether further efforts or future developments are underway or planned to advance preliminary evidence of this research**.

The discussion of the clinical implications has been improved (see Question I.4). We also added included a more complete answer regarding future device design in Question II.7.

ORIGINAL – Discussion -- Morphological description of AS patients

Such characterization might enrich the generation of synthetic AS geometries as the ones recently proposed by Verstraeten et al. (2024) for in-silico clinical trials.

AMENDED -- Discussion -- Morphological description of AS patients

Such enriched characterization might contribute to a more informed generation of detailed models of AS geometries for in-silico clinical trials - such as the one recently proposed by Verstraeten et al. (2024). Indeed, the SSM of the aorta and LVOT, the associated calcifications, and their relations to demographic and clinical variables, can be used to generate synthetic anatomies that reflect the morphological variability observed in a real patient population, helping both the design and testing of TAVI devices. The identified morphological traits (size, diameters, sinus and LVOT shape, calcium distribution) can also suggest features of importance for procedural planning, stressing common patterns of calcification that could impede proper valve expansion and sealing and would require specific care. Future studies will focus on quantifying the presented relationships and incorporating them into clinical decision-making processes and computational testing.

II.3. Section 5.3: "A base threshold of 300 Hounsfield unit was adapted to each image based on the operator assessment. We stress the fact that the focus of the study was on the spatial description of the calcification pattern and that better segmentations were favoured over consistent estimation of calcification scores."
Please clarify if CTA imaging was acquired with or without use of contrast agent, given its impact on voxels' HU intensity.
For the sake of reproducibility, the authors should clarify **what is meant by 'better segmentations were favoured' and provide details on how manual threshold corrections were managed**, including whether intra- and inter-operator variability was assessed.

We agree that more information regarding the image acquisition and segmentation was needed. Yes, contrast agent was used (Iomeron 400). More details on the segmentation process have also been added but we refer to Question I.1 for them.

ORIGINAL 5.2 Methods – Image processing

CTA images were acquired on a Siemens SOMATOM Force scanner. The acquisition was triggered and best-diastole images were used. The resolution was 0.4x0.4 mm and the slice thickness in the axial direction was 0.75 mm.

AMMENDED 5.2 Methods – Image processing

CTA images were routinely acquired on a Dual Source 90kVp Siemens SOMATOM Force scanner with contrast agent (Iomeron 400). The acquisition was triggered and best-diastole images were used. The slice thickness in the axial direction was 0.75 mm and the resolution was 0.4x0.4mm.

II.4. Section 5.5: "A vertical direction was manually aligned with the aortic root template main axis and used to defined two 2D cross-sectional slices. The first slice is 10mm-thick around the aortic valve (slice A on Figure 12), the second slice (slice B on Figure 12) is 20mm-thick above the first slice. The 3D calcification maps were then vertically averaged along in this direction for each slice.
The reported paragraph requires further attention. Neither slice A nor slice B is visible in Figure 12, which needs to be addressed. Additionally, the rationale for using 10mm-thick and 20mm-thick planes should be clarified to ensure the reader understands their scope.

The Figure referenced in this section should have been Figure 2 and not 12. We apologize for the mistake and corrected it. We also took the opportunity to do small edits of Figure 2 to clarify the definition of the two slices A and B.

Finally, the following sentence is modified to explain the choice of slice thickness:

ORIGINAL – 5.5 Methods – Spatial normalization of calcifications

A vertical direction was manually aligned with the aortic root template main axis and used to defined two 2D cross-sectional slices. The first slice is 10mm-thick around the aortic valve (slice A on Figure 12), the second one (slice B on Figure 12) is 20mm-thick above the first slice. The 3D calcification maps were then vertically averaged along in this direction for each slice.

AMMENDED – 5.5 Methods – Spatial normalization of calcifications

A vertical direction was manually aligned with the aortic root template main axis and used to defined two 2D cross-sectional slices. The first slice was designed around the aortic valve to include the complete leaflets and their calcifications (slice A on Figure 2.),

the second slice was designed above the first one to include most of the calcification on the ascending aortic wall (see slice B on Figure 2.). As a result, slice A was 10 mm thick, and slice B was 20 10 mm thick

---

II.5. Were **patients with bicuspid aortic valve** (BAV) enrolled in the study?

Yes, 3 patients have been identified as having BAV prior to the intervention. We however want to note that other patients may have had merged leaflets due to calcification but the observation is then difficult.

ADDED -- 2.1.1 Results – Population characteristics – Demographic and clinical information

Three patients with bicuspid aortic valves were identified.

II.6. A total of 130 patients were enrolled. **How did the authors determine whether this sample size was sufficient** for the analysis using statistical shape analysis?

This study does not have one (or a set of) clearly defined outcome(s). It is not possible to calculate a single power or sample size in the conventional manner, even if it does not mean that it cannot provide valuable insights. We clearly do not have the power to rigorously perform all the analyses that we did, and the objective was a description of the population "as accurate as possible" (but to this regard I think the absence of control of eventual recruitment bias, in particular due to the single centre limitation, may even be more critical). It is, in some ways, a preliminary study that could be used, I hope, to estimate required sample sizes in future ones. In this context, the number of patients was based on similar studies and on practical consideration (time to collect and segment the data).

We can however (ballpark) estimate that with N=130 subjects, the accuracy of the mean estimate d, in relation to the standard deviation s is:
*d = sqrt(1/N) * Z_alpha * s = 0.17 s (so around one fifth of a s.d.) for a Gaussian variable at alpha=0.05 level.*
Similarly difference between two equally sized sub-groups can be detected up to:
*d = sqrt(2/N) * (Z_alpha + Z_beta) * s = 0.35 s (so around one third of a s.d.) for a Gaussian variable at alpha=0.05 level and power beta=0.80.*
This can, in particular, be applied to the shape model parameters, keeping in mind that the standard deviation may vary from point to point (and the same question for the global statistic in the discriminant analysis would be a bit more complex).

We add a short sentence for the reasoning behind the number of included patients.

ORIGINAL 5.1 Methods -- Data and participants

Clinical data and images from 130 patients were retrospectively included in this study.

AMMENDED 5.1 Methods -- Data and participants

The sample size objective was based on similar studies, number of patients available, and practical considerations (time to collect and segment the data). Clinical data and images from 130 patients were retrospectively included in this study.

II.7. How "the presented morphological characterization [...] could contribute to [...] plan future improvement of TAVI device design" (as highlighted at the end of the abstract) should be made clearer to the reader.

As mentioned to Reviewer 1, we acknowledge the Reviewer's comment that the insights for device manufacturers might not appear too direct and rather focused on the morphological description of patients undergoing TAVI. However, the enriched morphological analysis can speak to clinicians (to predict outcomes), device manufacturers (to takes into account specific features of the implantation site) or both to conduct stratified statistical analysis including the so-called in silico trials. This can be particularly relevant if the morphological characterization takes into account a wider range of factors – such as, for example. the complexity of inter-individual variability and the presence of relevant comorbidities.

We agree with the Reviewer that this is mainly a future direction and, therefore, a specific point of discussion has been added to the Discussion section.

ORIGINAL – Abstract

In conclusion, the presented morphological characterization of patients with AS could contribute to understand TAVI outcomes and plan future improvement of TAVI device design.

AMENDED – Abstract

In the future, the presented morphological characterization of patients with AS could contribute to predict post-TAVI PVL and design and test improved TAVI devices.

ORIGINAL – Discussion -- Morphological description of AS patients

Such characterization might enrich the generation of synthetic AS geometries as the ones recently proposed by Verstraeten et al. (2024) for in-silico clinical trials.

AMENDED -- Discussion -- Morphological description of AS patients

Such enriched characterization might contribute to a more informed generation of detailed models of AS geometries for in-silico clinical trials - such as the one recently proposed by Verstraeten et al. (2024). Indeed, the SSM of the aorta and LVOT, the associated calcifications, and their relations to demographic and clinical variables, can

be used to generate synthetic anatomies that reflect the morphological variability observed in a real patient population, helping both the design and testing of TAVI devices. The identified morphological traits (size, diameters, sinus and LVOT shape, calcium distribution) can also suggest features of importance for procedural planning, stressing common patterns of calcification that could impede proper valve expansion and sealing and would require specific care. Future studies will focus on quantifying the presented relationships and incorporating them into clinical decision-making processes and computational testing.

II.8. Table 3: The authors are encouraged to **comment on the rates reported for mild post-TAVI AR** (78/124, 62.9%) and mild post-TAVI PVL (49/115, 42.6%). Including the percentage values directly in the table is also highly recommended for clarity and ease of interpretation.

We added the corresponding percentage to Table 3. A comment was also included to compare the PVL incidence rate to other studies. Limitations of the study in this regard is the mix of implanted devices (that was already mentioned) but also the lack of long-term post-discharge follow-up. In particular the rate of PVL is known to decrease over time after the intervention. We added on sentence in the discussion to mention it.

ADDED -- 2.4.1 Results – Prediction of interventional outcomes – Outcome assessment

The PVL incidence (mild-to-severe) of 53.9% is comparable to previously published results such as Gilbert et al. (2018) reporting a 43.2% pre-discharge PVL incidence for Edward Sapien valves (50.5% for Edward valves only in our population) and of 56.7% for Medtronic core valves.

ADDED -- Discussion

A future step would be to gather 1-year follow-up data regarding long-term PVL, complications, readmission, and quality of life changes

---

II.9. In the discussion, the authors highlight the novelty of the study in terms of the morphological characterization of the implantation site in patients undergoing TAVI (as well as in the conclusion, i.e., “new methodology to standardize and compare segmented 3D calcification”). While the potential of the present analysis is acknowledged, the authors should further elaborate on and critically **discuss the novelty of their approach, particularly in the context of prior research** focusing on the pre-TAVI assessment of aortic anatomy.

The methodological novelty is two-fold. First, 3D shape models of the aorta (or of the aortic root) are common but they have never been used in the context of TAVI outcome prediction. It complements both subject-specific modelling and features such as diameters (mainly the annulus), or vessel eccentricity (Schuhbakeck2015) or curvature (Gorla2024) etc. that have been assessed before. Verstraeten 2024 proposed a shape model of a TAVI population but with the objective of creating synthetic cohorts. Second, the 3D statistical model of calcification is, to the extend of our knowledge, novel. Previously, calcification assessments were at best measured by area (in each sinus etc.), not at a voxel level with spatial correspondence established between every subject in a population.
It has been included in the discussion.

ORIGINAL Discussion

Our study presented a novel, morphological characterization of the implantation site in patients diagnosed with aortic stenosis who were referred for TAVI procedures. It also explored the relationships between morphological variations and clinical findings both before and after the procedure.

Morphological description of AS patients. The proposed 3D non-parametric description allowed to distinguish variations of the aortic root and LVOT in terms of size, diameters, angulation and sinus shape. […] the 3D calcification model, achieved for the first time using shape analysis, allowed for a fine statistical description of the calcification that is not dependant on a difficult valve segmentation.

AMMENDED Discussion

Our study presented a complete morphological characterization of the implantation site in patients diagnosed with aortic stenosis who were referred for TAVI procedures. It also explored the relationships between morphological variations and clinical findings both before and after the procedure.

Morphological description of AS patients. The proposed 3D non-parametric description allowed to distinguish variations of the aortic root and LVOT in terms of size, diameters, angulation and sinus shape beyond expertly designed scalar parameters (Schuhbaeck2015, Gorla2024). […] the 3D calcification model, achieved for the first time using shape analysis, allowed for a fine statistical description of the calcification that is not dependent on a difficult valve segmentation.

II.10. Page 14: “We have, for example, seen how the amount of calcification impacted the choice of device and size with a preference for balloon-inflatable, smaller devices in highly calcified cases in order to limit the intervention risk.” This confirms well-known evidence from

previous clinical practice. It is worth noting that **only a small cohort of patients** (17/110, 15.5%) in this study was implanted with and investigated **using self-expandable devices**.

We agree it was a confirmation of what was reported about the usual practice by the clinicians in our team. The limitation including the device set has been made clearer in the limitations. his is clarified in the discussion, and a reference is added regarding the effect of calcification on device choice.

ORIGINAL Discussion – Study limitations.

The conclusions of our study are based on a cohort of patients retrospectively recruited from a single centre which might not fully representative of the AS population undergoing TAVI. In this context, there is not a homogeneous distribution of TAVI devices commonly used to perform the intervention.

AMMENDED Discussion – Study limitations.

The conclusions of our study are based on a cohort of patients retrospectively recruited from a single centre which might not fully be representative of the AS population undergoing TAVI.

In particular, the focus of this study was not on comparative device performance, or studying the most recent devices improvements; and in this context, there was a heterogeneous distribution of TAVI devices with self-inflating devices poorly represented in our sample (only 17/110, 15.5%). A wider multi-centre study covering more recent devices would provide additional insights.
